# Screening key microRNAs for castration-resistant prostate cancer based on miRNA/mRNA functional synergistic network

**DOI:** 10.18632/oncotarget.6102

**Published:** 2015-10-31

**Authors:** Jin Zhu, Sugui Wang, Wenyu Zhang, Junyi Qiu, Yuxi Shan, Dongrong Yang, Bairong Shen

**Affiliations:** ^1^ Department of Urology, Second Affiliated Hospital of Soochow University, Suzhou, China; ^2^ Department of Urology, Huai'an Hospital Affiliated to Xuzhou Medical College and Second People's Hospital of Huai'an, Huai'an, China; ^3^ Center for Systems Biology, Soochow University, Suzhou, China; ^4^ Department of General Surgery, The First Affiliated Hospital of Soochow University, Suzhou, China

**Keywords:** castration-resistant prostate cancer, microRNA biomarker, microRNA-mRNA interaction, bioinformatics model, pathway analysis

## Abstract

High-throughput methods have been used to explore the mechanisms by which androgen-sensitive prostate cancer (ASPC) develops into castration-resistant prostate cancer (CRPC). However, it is difficult to interpret cryptic results by routine experimental methods. In this study, we performed systematic and integrative analysis to detect key miRNAs that contribute to CRPC development. From three DNA microarray datasets, we retrieved 11 outlier microRNAs (miRNAs) that had expression discrepancies between ASPC and CRPC using a specific algorithm. Two of the miRNAs (miR-125b and miR-124) have previously been shown to be related to CRPC. Seven out of the other nine miRNAs were confirmed by quantitative PCR (Q-PCR) analysis. MiR-210, miR-218, miR-346, miR-197, and miR-149 were found to be over-expressed, while miR-122, miR-145, and let-7b were under-expressed in CRPC cell lines. GO and KEGG pathway analyses revealed that miR-218, miR-197, miR-145, miR-122, and let-7b, along with their target genes, were found to be involved in the PI3K and AKT3 signaling network, which is known to contribute to CRPC development. We then chose five miRNAs to verify the accuracy of the analysis. The target genes of each miRNA were altered significantly upon transfection of specific miRNA mimics in the C4–2 CRPC cell line, which was consistent with our pathway analysis results. Finally, we hypothesized that miR-218, miR-145, miR-197, miR-149, miR-122, and let-7b may contribute to the development of CRPC through the influence of Ras, Rho proteins, and the SCF complex. Further investigation is needed to verify the functions of the identified novel pathways in CRPC development.

## INTRODUCTION

Prostate cancer (PC) is the most commonly diagnosed malignancy and the second leading cause of cancer-related death in men in western countries [1–3]. The incidence and mortality rates of PC have increased dramatically in Asia in the last decade [4–6]. Patients who are not suitable for radical therapy are often treated with androgen deprivation therapy (ADT) and initially have a good response. However, nearly all of these patients eventually progress from androgen-sensitive prostate cancer (ASPC) into castration-resistant prostate cancer (CRPC), which no longer responds to ADT and is characterized by local relapse and distant metastasis. Although alternative strategies such as chemotherapy and radiotherapy have some effect on these cases, they do not significantly improve survival [1, 7, 8].

Mechanism studies indicate that androgen receptor (AR)-related signal pathways play important roles in CRPC development [9]. However, under castration, other pathways such as growth factors [10–14], *Bcl-2* [15], and the Akt pathway [14, 16] can stimulate PC growth. This indicates that the process from ASPC to CRPC involves sophisticated networks including multiple genes and pathways. New methods, other than those currently provided that focus on single genes, are required to investigate interactions between multiple signalling pathways [17–23].

MicroRNAs (miRNAs), a class of small non-coding RNAs approximately 22 nucleotides in length, are known to play important regulatory roles through protein translation inhibition and/or targeting mRNA cleavage [24, 25]. MiRNAs are involved not only in normal cellular growth, differentiation, and apoptosis [26–28], but also in cancer development and progression [29–32].

Several genome-wide miRNA expression profile studies have been used to identify PC-specific miRNA signatures [30, 33–35]. In these studies, it has been found that miRNAs are differentially expressed in ASPC as compared to CRPC cell lines [33, 34]. MiRNAs contribute to CRPC development by regulating *Bcl-2*, AKT, or mTORs [36–38]. However, how miRNAs regulate these pathways is largely unknown.

**Figure 1:**
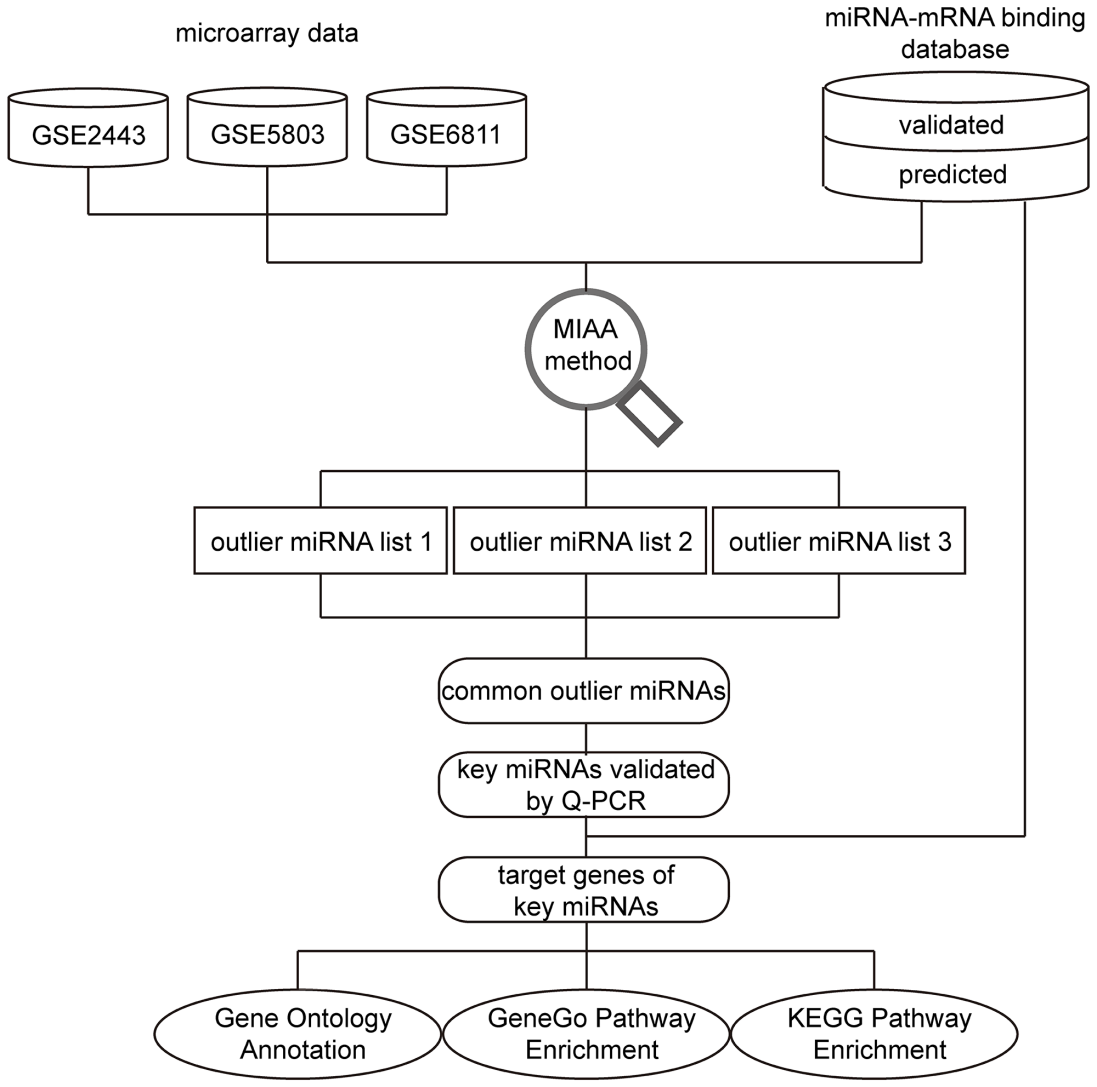
Schematic of the data analysis pipeline Gene expression profiles from three microarray datasets and miRNA-mRNA interaction data were combined as input data for further analysis with miRNA activity analysis (MIAA). After implementation of MIAA, three outlier miRNA lists were obtained. Subsequently, 11 outlier miRNAs were selected for follow-up by confirming their expression discrepancies in different PC cell lines by Q-PCR. These experimentally validated miRNAs are referred as “real” key miRNAs. Finally, target genes of these outlier miRNAs were further analyzed by GO annotation, KEGG and GeneGo pathway enrichment analysis, and miRNA-mRNA interaction network construction.

In this study, we used a novel method called miRNA activity analysis (MIAA), which combines gene expression data with miRNA/mRNA interaction data, to identify condition-specific miRNA activity and to predict miRNA expression status and involved pathways in CRPC development. MIAA was first used to identify CRPC-related miRNA activity. We found that several miRNAs have an expression difference between CRPC and ASPC cell lines. Additionally, some of the related pathways have not yet been reported. The schematic pipeline including procedures for data analysis is presented in Figure 1.

## RESULTS

### Differentially expressed miRNAs between ASPC and CRPC

In the MIAA study, when the Z-score threshold was set as 0.1, a total of 643 miRNAs were found to be differentially expressed in the three selected datasets. We then considered the top 5% (34 of 643, 5.3%) of miRNAs that had the highest Z-score as potential “real” key miRNAs. Among these 34 extracted miRNAs, 14 miRNAs showed an expression difference between any two datasets and 11 miRNAs showed a difference in all three datasets (Supplementary Table S1 and Figure S1). Therefore, we selected these 11 miRNAs as the most interesting miRNAs for further analysis. Among them, two miRNAs (miR-125b and miR-124) have been reported to be involved in the development of CRPC [33, 39], while the other nine miRNAs have not yet been reported to have expression differences between ASPC and CRPC. Thus, we decided to use a quantitative reverse transcription PCR (Q-PCR) assay to detect the expression status of these nine miRNAs in different PC cell lines. Another two extracted miRNAs (miRNA-585 and miRNA-149) which had the highest Z-score and were expressed in two datasets were also tested.

### The majority of the selected miRNAs were experimentally verified to have expression differences between ASPC and CRPC

The expression of the 11 selected miRNAs, miR-1, miR-210, miR-218, miR-155, miR-145, miR-122, miR-197, miR-346, let-7b, miR-149, and miR-585, were then experimentally verified by Q-PCR in the ASPC cell line LNCaP versus the CRPC cell lines C4–2, CWR22Rv1, PC-3, and DU145.

**Figure 2:**
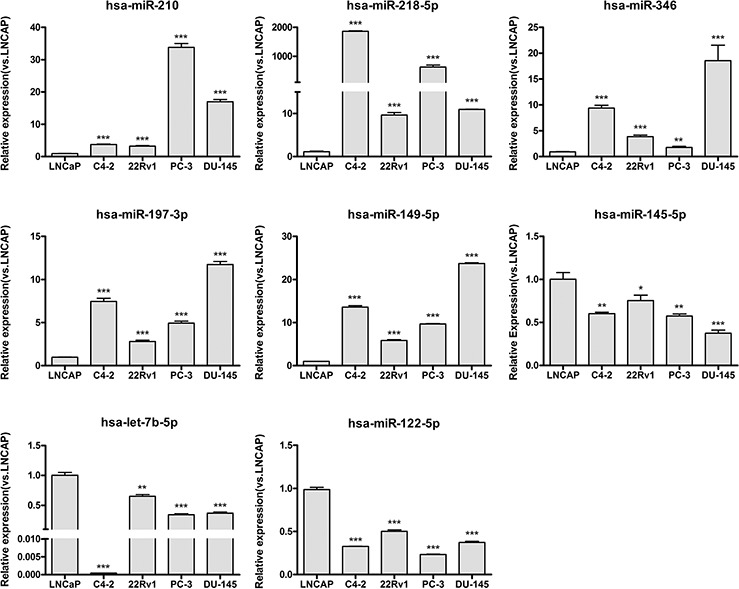
Experimental verification of expression differences for the screened miRNAs between ASPC and CRPC cell lines The expression status of 8 miRNAs, miR-210, miR-197–3p, miR-149–5p, miR-346, miR-218–5p, let-7b-5p, miR-145–5p, and miR-122–5p, was determined in LNCap, C4–2, CWR22Rv1, PC-3, and DU-145 human PC cell lines. The expression status of these miRNAs was normalized against U6 snRNA expression. Data are represented as the mean ± SD of three biological and three technical replicates. *P < 0.05, **P < 0.01, ***P < 0.001.

As the results in Figure 2 show, 8 of the 11 tested miRNAs (73%) were confirmed to have an expression difference between the ASPC and CRPC cell lines. Among these eight miRNAs, miR-210, miR-218, miR-346, miR-197, and miR-149 were up-regulated, while miR-122, miR-145, and let-7b were down-regulated in the CRPC cell lines. Expression of the other three (miRNA-1, miRNA-155, and miRNA-585) did not show statistically significant differences (data not shown). Along with miR-125b and miR-124, which were confirmed in published data [33, 39], 10 candidate miRNAs that were predicted by our MIAA model have been shown to have expression differences between ASPC and CRPC cell lines. However, except for miR-125b and miR-124, how these eight miRNAs are involved in pathways that contribute to CRPC transformation has not yet been reported.

### Integrative analysis revealed that novel miRNAs are involved in biological networks related to CRPC transformation

We performed Gene Ontology (GO) analysis, pathway enrichment analysis, and miRNA-mRNA interaction network construction analysis to investigate how these MIAA-derived and Q-PCR confirmed key miRNAs affect CRPC development.

**Figure 3:**
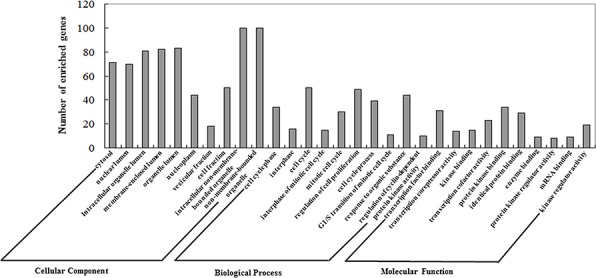
Gene Ontology (GO) annotation of genes regulated by confirmed key miRNAs Genes regulated by experimentally confirmed outlier miRNAs were retrieved and annotated with DAVID for the categories molecular function, biological process, and cellular component. The top 10 significantly enriched items for each feature are presented.

The GO annotation results are summarized at three different levels, and the 10 most highly enriched items for each domain are presented in Figure 3. We found that these miRNAs were correlated with several CRPC-associated biological processes [40].

**Figure 4:**
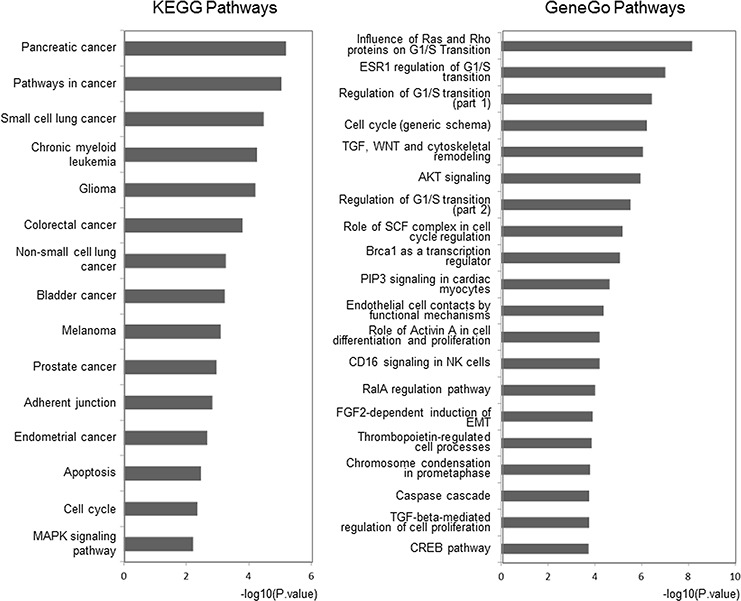
Pathway analysis for involved genes regulated by confirmed key miRNAs The outlier genes regulated by experimentally confirmed outlier miRNAs were retrieved and enriched in KEGG and GeneGo pathways with DAVID and GeneGo, respectively. The statistical significance level (*P*-value) was negative 10-based log transformed. The top 15 and 20 significantly enriched pathways for the KEGG database and the GeneGo database, respectively, are listed. Among the GeneGo pathways, seven pathways (marked with *) were previously shown to be related to CRPC development.

Meanwhile, we utilized the Database for Annotation, Visualization and Integrated Discovery (DAVID) and MetaCore™ to map these outlier miRNAs to KEGG and GeneGo pathways (see Supplementary Table S2 and Table S3). The top 15 highly enriched KEGG pathways, which included AKT signaling, the cell cycle, and apoptosis pathways, were plotted (Figure 4, left panel).

In the 20 most highly enriched pathways from the GeneGo Database (Figure 4, right panel), we found that these outlier miRNAs involved pathways, including AKT signaling, the cell cycle (including the Skp1-cullin-F-box ubiquitin ligase (SCF) complex), and proteins related to the G1/S transition (including Ras and Rho proteins), were enriched in a series of cancers, especially in PC. Seven pathways (including the cell cycle and AKT signaling pathway) have previously been reported to contribute to CRPC development [40–44] (Supplementary Table S4). Thus, we believe that the other 13 novel pathways could also be potential key pathways that contribute to CRPC development. However, their functions have not yet been reported and need further clarifying.

**Figure 5:**
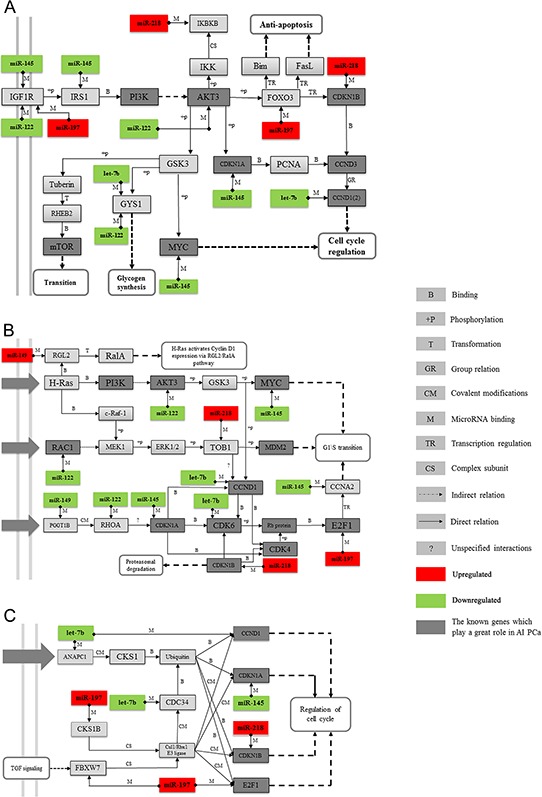
Examples of key miRNAs and their related pathways Three enriched GeneGo pathways from Figure 4 were retrieved and reconstructed, and the miRNA-mRNA regulation pairs were also appended. **A.** The AKT signaling pathway. The target genes and their related network of miR-218, miR-197, miR-145, miR-122, and let-7b were found to consist of PI3K and the AKT3 signaling network, which is part of the AKT signaling pathway. **B.** Influence of Ras and Rho proteins on the G1/S Transition. **C.** The role of the SCF complex in cell cycle regulation. MiR-218, miR-145, miR-197, miR-149, miR-122, and let-7b were indicated to influence the function of Ras and Rho proteins and the SCF complex and thus could contribute to the development of CRPC. **D.** The legend for the symbols included in these pathway maps.

The AKT pathway is correlated with PC progression, so we first chose the AKT pathway as our target to construct an miRNA-mRNA interaction network to investigate how miRNAs regulate their target gene. The results showed that miR-218, miR-197, miR-145, miR-122, and let-7b, along with their target genes, fit well into the PI3K and AKT3 signal networks, which are parts of the AKT signal pathway (Figure 5A). We then constructed two novel pathways, which consisted of miR-218, miR-197, miR-145, miR-122, let-7b, and miR-149. Our results showed that they may also contribute to the development of CRPC through their target gene *Ras*, Rho proteins (Figure 5B), and the SCF complex (Figure 5C). However, how these outlier miRNAs regulate Rho proteins and the SCF complex, which in turn regulate PC progression, has not yet been studied.

### Experimentally spot checking of key miRNAs in CRPC cells

**Figure 6:**
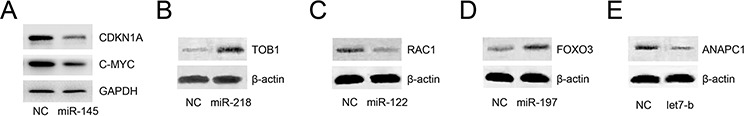
Experimental spot checking of key miRNAs in CRPC cells Among the 8 key miRNAs, miR-145, miR-218, miR-122, miR-197, and let7b were selected to check their roles in CRPC. C4–2 cells were transfected with either miRNA mimics or negative control. Western blot analyses revealed that upon transfection of miRNA mimics, their target proteins were altered (increased or decreased) significantly **A–E**.

Since all the abovementioned functions of key miRNAs were inferred *in silico*, we checked whether these miRNAs could inhibit mapped target genes *in vitro*. We noticed that miR-145 is the most widely studied miRNA in cancer, especially in PC, and our analysis showed that it could inhibit the expression of c-MYC and CDKNIA, which play important roles in CRPC. However, whether miR-145 could inhibit CDKNIA expression has not been reported in PC, so we chose miR145 as an interesting target for analysis. CRPC C4–2 cells were transfected with either miR-145 or negative control mimics. Western blot analyses revealed that upon transfection of miR-145, these two proteins were notably inhibited (Figure 6A).

Furthermore, we randomly chose four additional miRNAs (miR-218, miR-122, miR-197, and let7b) and one target gene of each of these miRNAs according to the constructed pathway map. CRPC C4-2 cells were transfected with either miRNA mimics or negative control mimics. Western blot analyses revealed that upon transfection of miR-218, miR-122, miR-197, or let7b, their target proteins were altered significantly (Figure 6B–6E), results that are in accordance with the constructed map in Figure 5.

## DISCUSSION

The mechanism of the development of castration-resistance has been proposed to be largely related to AR itself or AR-regulated genes. As for the *AR* gene, the mechanism may be via gene amplification, mutations, epigenetic modification of *AR* activity, splice variants, or co-activators/co-repressors that stimulate CRPC cell growth [45]. As for AR-regulated genes, abnormal expression of these genes under castration could stimulate CRPC cell growth by modifying cell proliferation, the cell cycle, or apoptosis [46]. Current strategies to fight CRPC either focus on targeting AR itself or its downstream genes [47]. A third potential strategy could be targeting the miRNAs noted in this study, which serve as bridges between AR and downstream target genes or non-AR pathways including Akt pathways. However, the key miRNAs that are responsible for the development of ASPC into CRPC are still largely unclear.

The ideal method for screening CPRC-related miRNAs is to perform miRNA microarray analysis using two prostate tissue samples, one from ASPC and the other from CRPC, from the same patient. However, such tissue samples are difficult to obtain. Thus, published studies usually use ASPC vs. CRPC cell lines for miRNA microarray analysis [33, 34]. However, these studies cannot fully reflect the miRNA changes in PC patients since PC tumors are heterogenic. Although several microarray analyses have focused on differential gene expression between different human ASPC and CRPC samples, the results derived from these assays, especially from genes presumed to be down- or up-regulated, can be quite cryptic and require intensive follow-up studies to confirm the function of candidate genes one by one with traditional experiments. Therefore, using new methods that can predict the function of candidate genes prior to experimental analysis may save time and effort by indicating what should be prioritized for further study. These considerations prompted us to screen key miRNAs correlated with CRPC using bioinformatics methods.

In this study, we retrieved gene expression information from three DNA microarray datasets and extracted the top 5% significantly changed miRNAs for further study. Our results showed that 11 miRNAs were differentially expressed between ASPC and CRPC cell lines. Two of the 11 retrieved miRNAs, miRNA-125b [35, 48] and miRNA-124 [49], have been shown to be up-regulated in CRPC cell lines. Nine miRNAs which have not been investigated in CRPC previously were confirmed to have expression differences between ASPC and CRPC cell lines in our experiments. The fact that miR-218, miR-149, and miR-145 have been found to be differentially expressed in a number of tumors [50, 51] suggests that they may have important roles in the development of CRPC.

Using GO analysis [52], we found that these validated miRNAs were correlated with several CRPC-associated biological processes, such as the cell cycle and AKT signaling [6, 16, 41]. We then applied pathway analysis and regulatory network analysis using the KEGG database, which contains the current knowledge on gene and molecular interactions. We found that these outlier miRNAs involved pathways that were enriched in PC. The cell cycle [53, 54] and MAPK signaling pathways [55] may play crucial roles in the development of CRPC, and some cell cycle-related genes, such as *cyclin G1*, *cyclin D1*, and *cyclin D3*, can be regulated by miRNA-122 and let-7b [54, 56], which provide additional evidence that the results of our MIAA analysis are truly correlated with CRPC development.

Since miR-145 is predicted to be a key miRNA and is down-regulated in CRPC, theoretically it should be a tumor suppressor in CRPC. A western blot assay showed that miR-145 targets c-MYC and CDKN1A, which is consistent with the constructed map. C-MYC is an overexpressed oncogene in PC [57] and has been reported to promote the progression of CRPC [58]. Interestingly, although this finding is consistent with the constructed pathway map, CDKN1A induces cell cycle arrest, which is contrary to our hypothesis, and a previously study showed that miR-145 induces cell cycle arrest in PC [59]. Another study showed that miR-145 does inhibit CDKN1A, but also induces major pro-apoptosis molecules. Consequently, the response of the anti-proliferation effect of miR-145 is mainly due to apoptosis rather than cell cycle arrest [60]. This finding reflects the sophisticated miRNA/mRNA interaction network in CRPC.

On the other hand, Ras has been reported to contribute to the castration-resistance transformation of PC [61]. Along with its oncogenic role in other cancers, Ras was once regarded as a therapeutic target of interest. However, no agent has yet been developed to target it [62]. Modulating upstream miRNAs to target Ras might be a better choice. Rho is involved in AR activation and cell invasion in PC [63–67]. The SCF complex is involved in the cell cycle, apoptosis, and signaling pathways in several cancers [68, 69]. Our analysis results also indicated that miR-218, miR-197, miR-145, miR-122, let-7b, and miR-149 might contribute to the development of CRPC through their target genes Ras, Rho, and the SCF complex. However, how these miRNAs regulate the function of these targets in CRPC development is still largely unknown. Our findings suggest that these should be followed up in further experiments.

In summary, we used the MIAA method to extract miRNAs that correlated with CRPC. We confirmed several miRNAs that have expression discrepancies between ASPC and CRPC cell lines. We also investigated several novel pathways potentially involved in CRPC development. We believe that these predicted novel pathways should be prioritized for further study in the development of CRPC.

## MATERIALS AND METHODS

### Retrieval of PC gene expression microarray data

Publicly available datasets were retrieved from the Gene Expression Omnibus (GEO, http://www.ncbi.nlm.nih.gov/geo/) as raw data files. In order to locate microarray results from PC samples, “prostate cancer” and “DNA microarray” were used as search keywords. Then, we chose data derived from ASPC vs. CRPC samples. In total, three datasets, summarized in Table 1, could be used for further study [70–72].

**Table 1:** Summary of microarray datasets used in this study

GEO Accession	PMID	Platform	Probe Number	Number of samples		Ref#
				ASPC	CRPC	
GSE2443	16203770	Affymetrix Genome U133A Array	22283	10	10	31
GSE5803	17199135	Biostarit-141s	14112	18	8	32
GSE6811	17545589	YN Human 36K	36864	10	25	33

### Screening of key miRNAs with MIAA

MIAA is a simple but effective method previously developed to combine miRNA outlier activities with a disease diagnosis [31, 73, 74]. In the present study, it was applied to the analysis of gene expression discrepancies between the selected ASPC and CRPC datasets. The general procedures of MIAA were carried out as follows: (a) the human miRNA-mRNA target network was reconstructed by integrating the miRNA-mRNA binding data from 4 experimentally validated datasets (miRecords, Tarbase, miR2Disease, and miRTarbase) and 3 computationally predicted datasets (HOCTAR, ExprTargetDB, and starBase); (b) outlier genes were detected from the 3 abovementioned gene expression datasets; (c) a conditional sub-network was constructed for the intersection between outlier genes and the whole human miRNA-mRNA targeted network; and (d) the probability of outlier activity for each candidate miRNA was calculated using the following formula: Z-score = α/β, where α represents the number of outlier genes exclusively targeted by a specific miRNA and β represents the number of all outlier genes targeted by the same specific miRNA (α, β > 1).

All Z-score values of involved miRNAs were calculated. Only those miRNAs that presented with a higher Z-score than 0.1 were included as potential interesting outlier miRNAs. The outliers ranking in the top 5% were extracted as candidate miRNAs since numerous miRNAs were found to have expression differences in ASPC vs. CRPC cell lines.

### Cell culture

Five human PC cell lines, including one ASPC line (LNCaP) and four CRPC lines (C4-2, CWR22Rv1, PC-3, and DU-145), were used in this study. The cell lines were purchased from the American Type Culture Collection (Manassas, VA, USA) and maintained in RPMI-1640 media (Gibco) with 10% fetal bovine serum (Gibco), 1% streptomycin-penicillin (Invitrogen), and 1% L-glutamine (Invitrogen) at 37°C in a humidified atmosphere with 5% carbon dioxide.

### Q-PCR

Total RNA from cultured cells was extracted by TRizol reagent (Invitrogen, China). RNA (500 ng) was subjected to reverse transcription using the PrimeScript^®^ RT reagent kit (TaKaRa, China). MiRNA stem-loop primers were purchased from Guangzhou Ribo Bio Company. Q-PCR was performed using SYBR^®^ Premix Ex Taq™ (TaKaRa, China) on an Applied Biosystems 7500 real-time PCR machine (ABI). The relative expression of each miRNA was normalized against U6 snRNA. Each reaction was run at least in triplicate. Fold expression change was calculated according to the 2-ΔΔCt method [75].

### Functional synergistic analysis of key miRNAs

Genes targeted by these experimentally validated key miRNAs were retrieved first, followed by GO and pathway analysis to explore the function of these genes in the transformation from ASPC into CRPC. DAVID was used for GO annotation and KEGG pathway analysis at three levels: molecular function, biological process, and cellular component. The top ten highly enriched items for each domain are presented.

Another pathway source, MetaCore™, was used for GeneGo pathway mapping analysis. The significantly mapped pathways (*P*-Value < 0.05) were further confirmed via an NCBI PubMed literature exploration and miRNA-mRNA interaction network construction analysis. Finally, interesting miRNA-mRNA signal networks were investigated.

### miRNA mimics and transfection

For transient transfection, cells at 50% confluence were transfected with miRNA mimics or control mimics (Qiagen) using Lipofectamine3000 (Invitrogen) according to the manufacturer's instructions.

### Western blot analysis

Cells were washed with PBS and lysed in RIPA buffer. Proteins were separated on an SDS-PAGE gel and transferred to PVDF membranes (Millipore, Billerica, MA). The membranes were blocked in 5% non-fat milk in PBST for 1 h at room temperature and then incubated with diluted primary antibodies against GAPDH (Santa Cruz, #sc-166574), c-MYC (Cell Signaling, #9402S), CDKN1A (Cell Signaling, #2947P), β-Actin (Cell Signaling, #4970L) TOB1 (Proteintech, #14915–1-AP), RAC1 (Proteintech, #24072–1-AP), FOXO3 (Cell Signaling, #12829S), or ANAPC1(Proteintech, #21748–1-AP) overnight at 4°C. The blots were incubated with HRP conjugated secondary antibody for 1 h at room temperature, washed, and developed in the ECL system (Bio-Rad, Hercules, CA, USA).

### Statistical analysis

Graphpad Prism 5.0 software was used for data analysis. Data are presented as mean ± SD. Statistical analyses were performed using Student's *t*-test. The values were considered as statistically significant if the *P*-value was less than 0.05.

## SUPPLEMENTARY FIGURE AND TABLES



## References

[1] Schroder FH (2008). Progress in understanding androgen-independent prostate cancer (AIPC): a review of potential endocrine-mediated mechanisms. Eur Urol.

[2] Sirotnak FM, She Y, Khokhar NZ, Hayes P, Gerald W, Scher HI (2004). Microarray analysis of prostate cancer progression to reduced androgen dependence: studies in unique models contrasts early and late molecular events. Molecular carcinogenesis.

[3] Siegel RL, Miller KD, Jemal A (2015). Cancer statistics, 2015. CA: a cancer journal for clinicians.

[4] Chen XQ, Huang Y, Li X, Zhang P, Huang R, Xia J, Chen N, Wei Q, Zhu YC, Yang YR, Zeng H (2010). Efficacy of maximal androgen blockade versus castration alone in the treatment of advanced prostate cancer: a retrospective clinical experience from a Chinese medical centre. Asian journal of andrology.

[5] Qu YY, Dai B, Kong YY, Ye DW, Yao XD, Zhang SL, Zhang HL, Ma CG, Yang WY (2013). Prognostic factors in Chinese patients with metastatic castration-resistant prostate cancer treated with docetaxel-based chemotherapy. Asian journal of andrology.

[6] Chen J, Shao P, Cao Q, Li P, Li J, Cai H, Zhu J, Wang M, Zhang Z, Qin C, Yin C (2012). Genetic variations in a PTEN/AKT/mTOR axis and prostate cancer risk in a Chinese population. PloS one.

[7] Tammela T (2004). Endocrine treatment of prostate cancer. J Steroid Biochem Mol Biol.

[8] Yang DR, Ding XF, Luo J, Shan YX, Wang R, Lin SJ, Li G, Huang CK, Zhu J, Chen Y, Lee SO, Chang C (2013). Increased Chemosensitivity via Targeting Testicular Nuclear Receptor 4 (TR4)-Oct4-Interleukin 1 Receptor Antagonist (IL1Ra) Axis in Prostate Cancer CD133+ Stem/Progenitor Cells to Battle Prostate Cancer. The Journal of biological chemistry.

[9] Bluemn EG, Nelson PS (2012). The androgen/androgen receptor axis in prostate cancer. Current opinion in oncology.

[10] Feng S, Shao L, Yu W, Gavine P, Ittmann M (2012). Targeting fibroblast growth factor receptor signaling inhibits prostate cancer progression. Clinical cancer research : an official journal of the American Association for Cancer Research.

[11] Darrington E, Zhong M, Vo BH, Khan SA (2012). Vascular endothelial growth factor A, secreted in response to transforming growth factor-beta1 under hypoxic conditions, induces autocrine effects on migration of prostate cancer cells. Asian journal of andrology.

[12] Lamm ML, Long DD, Goodwin SM, Lee C (1997). Transforming growth factor-beta1 inhibits membrane association of protein kinase C alpha in a human prostate cancer cell line, PC3. Endocrinology.

[13] Al-Azayzih A, Gao F, Goc A, Somanath PR (2012). TGFbeta1 induces apoptosis in invasive prostate cancer and bladder cancer cells via Akt-independent, p38 MAPK and JNK/SAPK-mediated activation of caspases. Biochemical and biophysical research communications.

[14] Vo BT, Morton D, Komaragiri S, Millena AC, Leath C, Khan SA (2013). TGF-beta effects on prostate cancer cell migration and invasion are mediated by PGE2 through activation of PI3K/AKT/mTOR pathway. Endocrinology.

[15] Fleischmann A, Huland H, Mirlacher M, Wilczak W, Simon R, Erbersdobler A, Sauter G, Schlomm T (2012). Prognostic relevance of Bcl-2 overexpression in surgically treated prostate cancer is not caused by increased copy number or translocation of the gene. The Prostate.

[16] Floc'h N, Kinkade CW, Kobayashi T, Aytes A, Lefebvre C, Mitrofanova A, Cardiff RD, Califano A, Shen MM, Abate-Shen C (2012). Dual targeting of the Akt/mTOR signaling pathway inhibits castration-resistant prostate cancer in a genetically engineered mouse model. Cancer research.

[17] Jiang J, Jia P, Zhao Z, Shen B (2014). Key regulators in prostate cancer identified by co-expression module analysis. BMC genomics.

[18] Li Y, Vongsangnak W, Chen L, Shen B (2014). Integrative analysis reveals disease-associated genes and biomarkers for prostate cancer progression. BMC medical genomics.

[19] Jiang J, Jia P, Shen B, Zhao Z (2014). Top associated SNPs in prostate cancer are significantly enriched in cis-expression quantitative trait loci and at transcription factor binding sites. Oncotarget.

[20] Jiang J, Cui W, Vongsangnak W, Hu G, Shen B (2013). Post genome-wide association studies functional characterization of prostate cancer risk loci. BMC genomics.

[21] Chen J, Zhang D, Yan W, Yang D, Shen B (2013). Translational bioinformatics for diagnostic and prognostic prediction of prostate cancer in the next-generation sequencing era. BioMed research international.

[22] Chen J, Wang Y, Shen B, Zhang D (2013). Molecular signature of cancer at gene level or pathway level? Case studies of colorectal cancer and prostate cancer microarray data. Computational and mathematical methods in medicine.

[23] Wang Y, Chen J, Li Q, Wang H, Liu G, Jing Q, Shen B (2011). Identifying novel prostate cancer associated pathways based on integrative microarray data analysis. Computational Biology and Chemistry.

[24] He L, Hannon GJ (2004). MicroRNAs: small RNAs with a big role in gene regulation. Nat Rev Genet.

[25] Bartel DP (2009). MicroRNAs: target recognition and regulatory functions. Cell.

[26] Kloosterman WP, Plasterk RH (2006). The diverse functions of microRNAs in animal development and disease. Dev Cell.

[27] Baranwal S, Alahari SK (2010). miRNA control of tumor cell invasion and metastasis. Int J Cancer.

[28] Pang Y, Young CY, Yuan H (2010). MicroRNAs and prostate cancer. Acta Biochim Biophys Sin (Shanghai).

[29] Volinia S, Calin GA, Liu CG, Ambs S, Cimmino A, Petrocca F, Visone R, Iorio M, Roldo C, Ferracin M, Prueitt RL, Yanaihara N, Lanza G, Scarpa A, Vecchione A, Negrini M (2006). A microRNA expression signature of human solid tumors defines cancer gene targets. Proceedings of the National Academy of Sciences of the United States of America.

[30] Porkka KP, Pfeiffer MJ, Waltering KK, Vessella RL, Tammela TL, Visakorpi T (2007). MicroRNA expression profiling in prostate cancer. Cancer research.

[31] Zhang W, Zang J, Jing X, Sun Z, Yan W, Yang D, Guo F, Shen B (2014). Identification of candidate miRNA biomarkers from miRNA regulatory network with application to prostate cancer. J Transl Med.

[32] Tang Y, Yan W, Chen J, Luo C, Kaipia A, Shen B (2013). Identification of novel microRNA regulatory pathways associated with heterogeneous prostate cancer. BMC systems biology.

[33] Xu G, Wu J, Zhou L, Chen B, Sun Z, Zhao F, Tao Z (2010). Characterization of the small RNA transcriptomes of androgen dependent and independent prostate cancer cell line by deep sequencing. PloS one.

[34] Tang X, Tang X, Gal J, Kyprianou N, Zhu H, Tang G (2011). Detection of microRNAs in prostate cancer cells by microRNA array. Methods Mol Biol.

[35] Ozen M, Creighton CJ, Ozdemir M, Ittmann M (2008). Widespread deregulation of microRNA expression in human prostate cancer. Oncogene.

[36] Bitting RL, Armstrong AJ (2013). Targeting the PI3K/Akt/mTOR pathway in castration-resistant prostate cancer. Endocrine-related cancer.

[37] Du YF, Long QZ, Shi Y, Liu XG, Li XD, Zeng J, Gong YG, Wang XY, He DL (2013). Prostate-targeted mTOR-shRNA inhibit prostate cancer cell growth in human tumor xenografts. International journal of clinical and experimental medicine.

[38] Hagman Z, Larne O, Edsjo A, Bjartell A, Ehrnstrom RA, Ulmert D, Lilja H, Ceder Y (2010). miR-34c is downregulated in prostate cancer and exerts tumor suppressive functions. Int J Cancer.

[39] Shi XB, Xue L, Yang J, Ma AH, Zhao J, Xu M, Tepper CG, Evans CP, Kung HJ, deVere White RW (2007). An androgen-regulated miRNA suppresses Bak1 expression and induces androgen-independent growth of prostate cancer cells. Proceedings of the National Academy of Sciences of the United States of America.

[40] Wang Q, Li W, Zhang Y, Yuan X, Xu K, Yu J, Chen Z, Beroukhim R, Wang H, Lupien M, Wu T, Regan MM, Meyer CA, Carroll JS, Manrai AK, Janne OA (2009). Androgen receptor regulates a distinct transcription program in androgen-independent prostate cancer. Cell.

[41] Ghosh PM, Malik S, Bedolla R, Kreisberg JI (2003). Akt in prostate cancer: possible role in androgen-independence. Current drug metabolism.

[42] Rinaldo F, Li J, Wang E, Muders M, Datta K (2007). RalA regulates vascular endothelial growth factor-C (VEGF-C) synthesis in prostate cancer cells during androgen ablation. Oncogene.

[43] Zhu ML, Partin JV, Bruckheimer EM, Strup SE, Kyprianou N (2008). TGF-beta signaling and androgen receptor status determine apoptotic cross-talk in human prostate cancer cells. The Prostate.

[44] Park MH, Lee HS, Lee CS, You ST, Kim DJ, Park BH, Kang MJ, Heo WD, Shin EY, Schwartz MA, Kim EG (2012). p21-Activated kinase 4 promotes prostate cancer progression through CREB. Oncogene.

[45] Tsao CK, Galsky MD, Small AC, Yee T, Oh WK (2012). Targeting the androgen receptor signalling axis in castration-resistant prostate cancer (CRPC). BJU international.

[46] Waltering KK, Helenius MA, Sahu B, Manni V, Linja MJ, Jänne OA, Visakorpi T (2009). Increased expression of androgen receptor sensitizes prostate cancer cells to low levels of androgens. Cancer research.

[47] Chen Y, Sawyers CL, Scher HI (2008). Targeting the androgen receptor pathway in prostate cancer. Current opinion in pharmacology.

[48] Shi XB, Xue L, Ma AH, Tepper CG, Kung HJ, White RW (2011). miR-125b promotes growth of prostate cancer xenograft tumor through targeting pro-apoptotic genes. The Prostate.

[49] Shi XB, Xue L, Ma AH, Tepper CG, Gandour-Edwards R, Kung HJ, Devere White RW (2012). Tumor suppressive miR-124 targets androgen receptor and inhibits proliferation of prostate cancer cells. Oncogene.

[50] Leite KR, Sousa-Canavez JM, Reis ST, Tomiyama AH, Camara-Lopes LH, Sanudo A, Antunes AA, Srougi M (2011). Change in expression of miR-let7c, miR-100, and miR-218 from high grade localized prostate cancer to metastasis. Urologic oncology.

[51] Schaefer A, Jung M, Mollenkopf HJ, Wagner I, Stephan C, Jentzmik F, Miller K, Lein M, Kristiansen G, Jung K (2010). Diagnostic and prognostic implications of microRNA profiling in prostate carcinoma. Int J Cancer.

[52] Faro A, Giordano D, Spampinato C (2012). Combining literature text mining with microarray data: advances for system biology modeling. Briefings in bioinformatics.

[53] Saito Y, Suzuki H, Matsuura M, Sato A, Kasai Y, Yamada K, Saito H, Hibi T (2011). MicroRNAs in Hepatobiliary and Pancreatic Cancers. Front Genet.

[54] Fornari F, Gramantieri L, Giovannini C, Veronese A, Ferracin M, Sabbioni S, Calin GA, Grazi GL, Croce CM, Tavolari S, Chieco P, Negrini M, Bolondi L (2009). MiR-122/cyclin G1 interaction modulates p53 activity and affects doxorubicin sensitivity of human hepatocarcinoma cells. Cancer research.

[55] Kinkade CW, Castillo-Martin M, Puzio-Kuter A, Yan J, Foster TH, Gao H, Sun Y, Ouyang X, Gerald WL, Cordon-Cardo C, Abate-Shen C (2008). Targeting AKT/mTOR and ERK MAPK signaling inhibits hormone-refractory prostate cancer in a preclinical mouse model. J Clin Invest.

[56] Schultz J, Lorenz P, Gross G, Ibrahim S, Kunz M (2008). MicroRNA let-7b targets important cell cycle molecules in malignant melanoma cells and interferes with anchorage-independent growth. Cell research.

[57] Buttyan R, Sawczuk IS, Benson MC, Siegal JD, Olsson CA (1987). Enhanced expression of the c-myc protooncogene in high-grade human prostate cancers. The Prostate.

[58] Gao L, Schwartzman J, Gibbs A, Lisac R, Kleinschmidt R, Wilmot B, Bottomly D, Coleman I, Nelson P, McWeeney S (2013). Androgen receptor promotes ligand-independent prostate cancer progression through c-Myc upregulation. PloS one.

[59] Zaman MS, Chen Y, Deng G, Shahryari V, Suh S, Saini S, Majid S, Liu J, Khatri G, Tanaka Y (2010). The functional significance of microRNA-145 in prostate cancer. British journal of cancer.

[60] Spizzo R, Nicoloso M, Lupini L, Lu Y, Fogarty J, Rossi S, Zagatti B, Fabbri M, Veronese A, Liu X (2010). miR-145 participates with TP53 in a death-promoting regulatory loop and targets estrogen receptor-α in human breast cancer cells. Cell Death & Differentiation.

[61] Bakin RE, Gioeli D, Bissonette EA, Weber MJ (2003). Attenuation of Ras signaling restores androgen sensitivity to hormone-refractory C4-2 prostate cancer cells. Cancer research.

[62] Ledford H (2015). Cancer: The Ras renaissance. Nature.

[63] Papadopoulou N, Charalampopoulos I, Alevizopoulos K, Gravanis A, Stournaras C (2008). Rho/ROCK/actin signaling regulates membrane androgen receptor induced apoptosis in prostate cancer cells. Experimental cell research.

[64] Sequeira L, Dubyk CW, Riesenberger TA, Cooper CR, van Golen KL (2008). Rho GTPases in PC-3 prostate cancer cell morphology, invasion and tumor cell diapedesis. Clinical & experimental metastasis.

[65] Wen S, Shang Z, Zhu S, Chang C, Niu Y (2013). Androgen receptor enhances entosis, a non-apoptotic cell death, through modulation of Rho/ROCK pathway in prostate cancer cells. The Prostate.

[66] Wu F, Peacock SO, Rao S, Lemmon SK, Burnstein KL (2013). Novel interaction between the co-chaperone Cdc37 and Rho GTPase exchange factor Vav3 promotes androgen receptor activity and prostate cancer growth. The Journal of biological chemistry.

[67] Somlyo AV, Bradshaw D, Ramos S, Murphy C, Myers CE, Somlyo AP (2000). Rho-kinase inhibitor retards migration and *in vivo* dissemination of human prostate cancer cells. Biochemical and biophysical research communications.

[68] Wei W, Ayad NG, Wan Y, Zhang GJ, Kirschner MW, Kaelin WG (2004). Degradation of the SCF component Skp2 in cell-cycle phase G1 by the anaphase-promoting complex. Nature.

[69] Yen HC, Elledge SJ (2008). Identification of SCF ubiquitin ligase substrates by global protein stability profiling. Science.

[70] Best CJ, Gillespie JW, Yi Y, Chandramouli GV, Perlmutter MA, Gathright Y, Erickson HS, Georgevich L, Tangrea MA, Duray PH, Gonzalez S, Velasco A, Linehan WM, Matusik RJ, Price DK, Figg WD (2005). Molecular alterations in primary prostate cancer after androgen ablation therapy. Clinical cancer research: an official journal of the American Association for Cancer Research.

[71] Wei Q, Li M, Fu X, Tang R, Na Y, Jiang M, Li Y (2007). Global analysis of differentially expressed genes in androgen-independent prostate cancer. Prostate cancer and prostatic diseases.

[72] Tamura K, Furihata M, Tsunoda T, Ashida S, Takata R, Obara W, Yoshioka H, Daigo Y, Nasu Y, Kumon H, Konaka H, Namiki M, Tozawa K, Kohri K, Tanji N, Yokoyama M (2007). Molecular features of hormone-refractory prostate cancer cells by genome-wide gene expression profiles. Cancer research.

[73] Chen J, Zhang D, Zhang W, Tang Y, Yan W, Guo L, Shen B (2013). Clear cell renal cell carcinoma associated microRNA expression signatures identified by an integrated bioinformatics analysis. J Transl Med.

[74] Huang J, Sun Z, Yan W, Zhu Y, Lin Y, Chen J, Shen B, Wang J (2014). Identification of microRNA as sepsis biomarker based on miRNAs regulatory network analysis. BioMed research international.

[75] Livak KJ, Schmittgen TD (2001). Analysis of relative gene expression data using real-time quantitative PCR and the 2(−Delta Delta C(T)) Method. Methods.

