# Supplementary material for: Screening key microRNAs for castration-resistant prostate cancer based on miRNA/mRNA functional synergistic network
Source: Oncotarget. 2015 Oct 31;6(41):43819–30. doi: 10.18632/oncotarget.6102 (PMC4791269; doi:10.18632/oncotarget.6102)
Supplement: Supplementary file 1 [file oncotarget-06-43819-s001.pdf]

SUPPLEMENTARY FIGURE AND TABLES

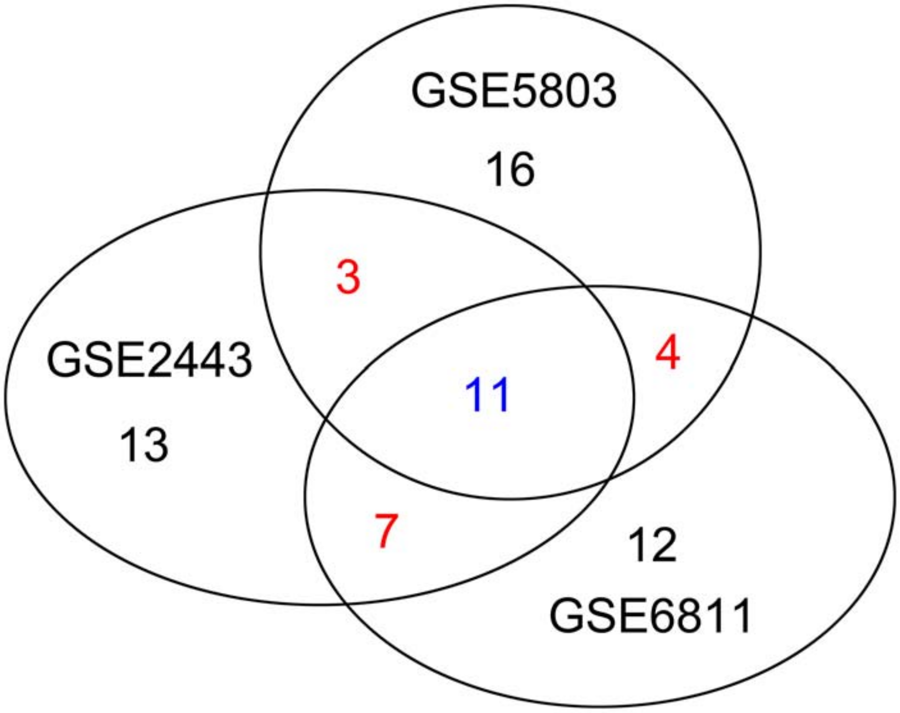

Supplementary Figure S1: Venn diagram illustrating the overlap in microRNA detection across the three data sets.

Supplementary Table S1: The predicted outlier miRNAs in each dataset

miRNA ID	GSE2443	GSE5803	GSE6811
hsa-miR-1	√	√	√
hsa-miR-210	√	√	√
hsa-miR-218(-5p)	√	√	√
hsa-miR-155	√	√	√
hsa-miR-145(-5p)	√	√	√
hsa-miR-122(-5p)	√	√	√
hsa-miR-197(-3p)	√	√	√
hsa-miR-346	√	√	√
hsa-let-7b(-5p)	√	√	√
hsa-miR-125b	√	√	√
hsa-miR-124	√	√	√
hsa-miR-149(-5p)	√		√
hsa-miR-585		√	√
hsa-miR-146a		√	√
hsa-miR-326	√	√	
hsa-miR-593	√	√	
hsa-miR-626	√	√	
hsa-miR-339(-3p)	√		√
hsa-miR-375	√		√
hsa-miR-192	√		√
hsa-miR-126	√		√
hsa-miR-627	√		√
hsa-miR-938	√		√
hsa-miR-490(-3p)		√	√
hsa-miR-378		√	√

Supplementary Table S2: Highly enriched (P-value< 0.05) KEGG pathways by target genes of 8 experimentally validated key miRNAs

	Pathway Term	Pathway Class	Subclass	P-Value
**1.**	Pancreatic cancer	Human Diseases	Cancers	6.44E-06
**2.**	Pathways in cancer	Human Diseases	Cancers	9.18E-06
**3.**	Small cell lung cancer	Human Diseases	Cancers	3.27E-05
**4.**	Chronic myeloid leukemia	Human Diseases	Cancers	5.47E-05
**5.**	Glioma	Human Diseases	Cancers	6.02E-05
**6.**	Colorectal cancer	Human Diseases	Cancers	1.57E-04
**7.**	Non-small cell lung cancer	Human Diseases	Cancers	5.44E-04
**8.**	Bladder cancer	Human Diseases	Cancers	5.85E-04
**9.**	Melanoma	Human Diseases	Cancers	8.00E-04
**10.**	Prostate cancer	Human Diseases	Cancers	1.08E-03
**11.**	Adherens junction	Cellular Processes	Cell Communication	1.44E-03
**12.**	Endometrial cancer	Human Diseases	Cancers	2.15E-03
**13.**	Apoptosis	Cellular Processes	Cell Growth and Death	3.39E-03
**14.**	Cell cycle	Cellular Processes	Cell Growth and Death	4.45E-03
**15.**	MAPK signaling pathway	Environmental Information Processing	Signal Transduction	5.95E-03
**16.**	p53 signaling pathway	Cellular Processes	Cell Growth and Death	9.71E-03
**17.**	Epithelial cell signaling in Helicobacter pylori infection	Human Diseases	Cardiovascular Diseases	1.60E-02
**18.**	Acute myeloid leukemia	Human Diseases	Cancers	1.60E-02
**19.**	Dilated cardiomyopathy	Human Diseases	Cardiovascular Diseases	1.73E-02
**20.**	Arrhythmogenic right ventricular cardiomyopathy (ARVC)	Human Diseases	Cardiovascular Diseases	2.51E-02
**21.**	Focal adhesion	Cellular Processes	Cell Communication	2.91E-02
**22.**	Endocytosis	Cellular Processes	Transport and Catabolism	3.00E-02
**23.**	Hypertrophic cardiomyopathy (HCM)	Human Diseases	Cardiovascular Diseases	3.35E-02
**24.**	TGF-beta signaling pathway	Environmental Information Processing	Signal Transduction	3.85E-02
**25.**	Viral myocarditis	Human Diseases	Cardiovascular Diseases	3.88E-02
**26.**	Regulation of actin cytoskeleton	Cellular Processes	Cell Communication	4.71E-02
**27.**	Tight junction	Cellular Processes	Cell Communication	4.71E-02
**28.**	Jak-STAT signaling pathway	Environmental Information Processing	Signal Transduction	4.86E-02
**29.**	B cell receptor signaling pathway	Organismal Systems	Immune System	4.89E-02

Supplementary Table S3: Top 50 GeneGo pathways enriched by target genes of 8 experimentally validated key miRNAs

	Pathway Term	Pathway Map Folder	*P*-Value
**1.**	Influence of Ras and Rho proteins on G1/S Transition	Cell cycle	6.68E-09
**2.**	ESR1 regulation of G1/S transition	Cell cycle	9.59E-08
**3.**	Regulation of G1/S transition (part 1)	Cell cycle	3.62E-07
**4.**	Cell cycle (generic schema)	Cell cycle	5.87E-07
**5.**	TGF, WNT and cytoskeletal remodeling	Cytoskeleton remodeling	8.68E-07
**6.**	AKT signaling	Signal transduction	1.12E-06
**7.**	Regulation of G1/S transition (part 2)	Cell cycle	2.97E-06
**8.**	Role of SCF complex in cell cycle regulation	Cell cycle	6.61E-06
**9.**	Brca1 as a transcription regulator	DNA damag	8.43E-06
**10.**	PIP3 signaling in cardiac myocytes	Development	2.26E-05
**11.**	Endothelial cell contacts by junctional mechanisms	Cell adhesion	4.09E-05
**12.**	Role of Activin A in cell differentiation and proliferation	Development	6.20E-05
**13.**	CD16 signaling in NK cells	Immune response	6.30E-05
**14.**	RalA regulation pathway	Cytoskeleton remodeling	9.68E-05
**15.**	FGF2-dependent induction of EMT	Development	1.22E-04
**16.**	Thrombopoietin-regulated cell processes	Development	1.35E-04
**17.**	Chromosome condensation in prometaphase	Cell cycle	1.57E-04
**18.**	Caspase cascade	Apoptosis and survival	1.69E-04
**19.**	Normal and pathological TGF-beta-mediated regulation of cell proliferation	Development	1.69E-04
**20.**	CREB pathway	Transcription	1.79E-04
**21.**	Macropinocytosis regulation by growth factors	Transport	1.96E-04
**22.**	LRRK2 and immune function in Parkinson's disease	Disease	2.00E-04
**23.**	Chemokines and adhesion	Cell adhesion	2.40E-04
**24.**	Regulation of actin cytoskeleton by Rho GTPases	Cytoskeleton remodeling	2.50E-04
**25.**	Cross-talk between Ras-family GTPases	G-protein signaling	2.50E-04
**26.**	Cytoskeleton remodeling	Cytoskeleton remodeling	2.83E-04
**27.**	VEGF signaling via VEGFR2 - generic cascades	Development	2.93E-04
**28.**	Endothelial cell contacts by non-junctional mechanisms	Cell adhesion	3.09E-04
**29.**	IGF-1 receptor signaling	Development	3.42E-04
**30.**	Nucleocytoplasmic transport of CDK/Cyclins	Cell cycle	3.50E-04
**31.**	K-RAS regulation pathway	G-protein signaling	3.78E-04

(*Continued*)

	Pathway Term	Pathway Map Folder	*P*-Value
**32.**	Role of HDAC and calcium/calmodulin-dependent kinase (CaMK) in control of skeletal myogenesis	Development	4.34E-04
**33.**	Prolactin receptor signaling	Development	6.75E-04
**34.**	Histamine H1 receptor signaling in the interruption of cell barrier integrity	Cell adhesion	9.62E-04
**35.**	Fibronectin-binding integrins in cell motility	Cytoskeleton remodeling	1.06E-03
**36.**	ACM regulation of nerve impulse	Neurophysiological process	1.08E-03
**37.**	MIF - the neuroendocrine-macrophage connector	Immune response	1.08E-03
**38.**	Alpha-1 adrenergic receptors signaling via cAMP	Development	1.22E-03
**39.**	PDGF signaling via STATs and NF-kB	Development	1.24E-03
**40.**	NF-AT signaling in Cardiac Hypertrophy	Cardiac Hypertrophy	1.34E-03
**41.**	Histamine H1 receptor signaling in immune response	Immune response	1.36E-03
**42.**	Integrin-mediated cell adhesion and migration	Cell adhesion	1.36E-03
**43.**	Inhibition of telomerase activity and cellular senescence	DNA damage	1.50E-03
**44.**	Oncostatin M signaling via JAK-Stat in human cells	Immune response	1.50E-03
**45.**	Integrin outside-in signaling	Cytoskeleton remodeling	1.51E-03
**46.**	Erk Interactions: Inhibition of Erk	Signal transduction	1.64E-03
**47.**	Cytoplasmic/mitochondrial transport of proapoptotic proteins Bid, Bmf and Bim	Apoptosis and survival	1.64E-03
**48.**	Some pathways of EMT in cancer cells	Development	1.87E-03
**49.**	EPO-induced Jak-STAT pathway	Development	1.87E-03
**50.**	Role of heterochromatin protein 1 (HP1) family in transcriptional silencing	Transcription	2.18E-03

Supplementary Table S4: Literature validated GeneGO pathways from enrichment of genes targeted by 8 key miRNAs

Pathway Term	Enrichment Genes	Enrichment miRNAs	PMID
Regulation of G1/S transition (part 1)	CDKN1B;FBXW7;CDKN1A;ZFYVE9; CCNA2;CCND2;ANAPC1;CCND1;CDK6	miR-218;miR-197;miR-145;let-7b	19646263;15216909
Cell cycle (generic schema)	E2F3;E2F1;CCNA2;CCND2;E2F5;E2F6; CCND1;CDK6	miR-210;miR-197;miR-145;let-7b	19632176;18097561
AKT signaling	IKBKB;CDKN1B;IGF1R;FOXO3;CDKN1A;IGF1R;IRS1;MYC;AKT3;GYS1; CCND2;CCND1	miR-218;miR-197;miR-145;miR-122;let-7b	14683476;21681193 21317204
Regulation of G1/S transition (part 2)	E2F1;CCNA2;AKT3;CCNA2;CCND2; CCND1;CDK6	miR-197;miR-145;miR-122;let-7b	19646263;15216909
RalA regulation pathway	ACTG1;RGL2;CALM1;HSF1;RALGDS; PGGTB;RAC1	miR-210;miR-149;miR-145;miR-122;let-7b	16964283
Normal and pathological TGF-beta mediated regulation of cell proliferation	ZFYVE9;MYC;RAC1;PDGFRA;CCND1	miR-145;miR-122;let-7b	18163430
CREB pathway	IGF1R;PRKAR2A;CACNA1D;CALM1; IRS1;RAC1;AKT3;CCND1	miR-197;miR-149;miR-145;miR-122;let-7b	22710715
